# A Preliminary Study on Transcriptional Regulation of SNP Site C-1888T in the Promoter Region of Human PLUNC Gene and Nasopharyngeal Carcinoma Susceptibility

**DOI:** 10.1155/genr/5148918

**Published:** 2024-12-19

**Authors:** Beina Liu, Rong Wang, Ying He

**Affiliations:** ^1^Department of Otolaryngology-Head and Neck Surgery, Sir Run Run Shaw Hospital, Zhejiang University School of Medicine, Hangzhou 310016, Zhejiang, China; ^2^Department of Otolaryngology-Head and Neck Surgery, Ruikang Hospital Affiliated to Guangxi University of Chinese Medicine, Nanning 530011, Guangxi, China; ^3^Department of Otolaryngology-Head and Neck Surgery, Nanfang Hospital, Nanfang Medical University, Guangzhou 510515, China

**Keywords:** nasopharyngeal carcinoma, PLUNC genes, single-nucleotide polymorphism, transcriptional regulation

## Abstract

**Purpose:** The transcriptional regulatory factors binding to the polymorphic site C-1888T in the promoter region of the palate, lung, and nasal epithelium clone (PLUNC) gene were identified to investigate whether the C-1888T polymorphic site affects the transcriptional regulation and function of PLUNC gene.

**Materials and Methods:** Three genotypes of C-1888T polymorphic locus were screened from established nasopharyngeal carcinoma (NPC) cells, and the mRNA expression levels of PLUNC gene in different genotypes were detected. The respective transcription factors that were more likely to bind with A or G in SNP were predicted by biological information and preliminarily verified in vitro by gel electrophoresis migration rate analysis. Ulteriorly, the NPC cell lines were analyzed through chromatin immunoprecipitation combined with PCR amplification to confirm that the transcription factors could bind to the PLUNC gene promoter.

**Results:** The cell lines 5–8F, 6–10B, CNE1, and CNE2 were heterozygous CT type, SUNE1 was homozygous CC type, and C666-1 was homozygous TT type. The expression of PLUNC gene was significantly different among all cell lines (*F* = 33.844, *p* < 0.001), and the gene expression level of CC type was significantly lower than TT type (*p* < 0.001). Gel electrophoresis mobility analysis confirmed that the transcription factors XFD3 and EVI1 could bind to the PLUNC gene promoter when the SNP was A and G, respectively. PCR amplification combined with chromatin immunoprecipitation showed that EVI1 could bind to the DNA fragment of the promoter region of PLUNC gene in SUNE1 NPC cells.

**Conclusion:** The transcription factors XFD3 and EVI1 may be involved in the transcriptional regulation of PLUNC gene, and EVI1 can bind to the promoter region of PLUNC gene in SUNE1 NPC cells, thus associated with the susceptibility/risk of NPC.

## 1. Introduction

Nasopharyngeal carcinoma (NPC) is a common malignant tumor in South China and Southeast Asia. The incidence of NPC is influenced by various factors, such as virus infection and environmental factors [1, 2]. Genetic susceptibility or predisposition is also considered a potential contributor to NPC development [3, 4]. As we know, the differences in susceptibility to certain diseases and drug responses of different populations and individuals are often determined by allelic polymorphisms of the related genes, mainly in the form of single-nucleotide polymorphisms (SNPs). However, SNPs located in coding, flanking, and regulatory regions may change gene function [5].

The PLUNC gene has emerged as a novel candidate for an antitumor gene [6], showing promising tissue-specific associations with NPC [7, 8]. Our previous study found that the two polymorphic loci C-2128T and C-1888T of PLUNC gene were closely related to the susceptibility of NPC in the Chinese population (OR = 2.8–3.3, *p* < 0.001). And the haplotype classification on this basis also showed that individuals with haplotype C-C were more likely to develop NPC (OR = 1.86, 95% CI = 1.34–2.56, *p*=0.00016) [9].

Wang analyzed the −5000 ∼ +1000 bp sequence at the 5′ end of PLUNC gene using the bioinformatics tool to predict that the promoter exists in the region from −490 bp to +89 bp. Based on these findings, it was confirmed that −240∼ +100 bp in the flanking region at the 5′ end of PLUNC gene had promoter activity through PCR amplification, luciferase vector construction, and identification [10, 11]. Precisely, the C-1888T polymorphism site is located in this region. The initial experiment demonstrated that the PLUNC gene promoter region SNP 1888TT genotype was capable of binding to nuclear proteins in human NPC CEN2 cells, whereas the CC genotype did not exhibit this binding capability [12]. Therefore, it is speculated that this polymorphic site affects the transcriptional regulation and function of the PLUNC gene, resulting in an increased susceptibility/risk of NPC. In this study, three types of these polymorphic loci were screened from the established NPC cells, and the mRNA expression levels of PLUNC gene in different genotypes were detected. At the same time, the transcriptional regulatory factors related to the polymorphic site C-1888T in the promoter region of PLUNC gene were preliminarily identified, which laid a foundation for further study on the regulation mechanism of PLUNC gene.

## 2. Materials and Methods

### 2.1. Materials

NPC cell lines 5–8F, 6–10B, CNE1, CNE2, and C666-1 were conventionally preserved in our laboratory, and cell line SUNE1 was provided by the Cancer Institute of Southern Medical University. The Cell Genome DNA Extraction Kit was purchased from Tiangen Biotech. Ex Tag enzyme, FbaI enzyme, Total RNA Extractant RNAiso Plus, Reverse Transcription Kit, and SYBRPremix Ex TagTM II (Perfect Real Time) Kit were purchased from Ta KaRa Co., Ltd. Nucleoprotein Extraction Kit and EMSA Probe Biotin Labeling Kit were purchased from Beyotime Biotechnology. LightShift Chemiluminescent EMSA Kit and Agarose ChIP Kit were purchased from PIERCE Company. The rabbit antihuman EVI1 antibody was purchased from SANTA CRUZ BIOTECHNOLOGY Company. The x-ray film is a product of Fuji Company. The vertical electrophoresis apparatus and electric transfer film apparatus are the products of Bio-Rad Company. The PCR instrument is a product of Eppendorf Company in Germany. The enzyme-linked immunosorbent assay model is BioTek ELx800. The probe synthesis and sequencing were completed by Shanghai Invitrogen Biotechnology Co., LTD.

### 2.2. Screening Three Genotypes of C-1888T Polymorphic Loci

DNA of NPC cells was extracted, and the full length 192 bp DNA sequence of the upstream promoter region of PLUNC gene was amplified by PCR-RFLP method. The PCR primer design, reaction system, and conditional were referenced [9]. 5 μL PCR product was digested by enzyme, and the reaction system and conditions were referenced [9]. PCR and enzyme-cutting products were analyzed with 3% agarose gel electrophoresis. The interpretation of results is as follows: after digestion, only 192 bp band was CC type, 170 bp and 22 bp bands were TT type, and all three bands were heterozygous CT type. At the same time, part of the PCR products were extracted and sequenced directly to verify the accuracy of enzyme digestion results.

### 2.3. Detect the Expression of PLUNC in Different Cell Lines Through Quantitative Real-Time PCR (QPCR)

Total RNA was extracted from NPC cell lines by RNAiso Plus, precipitated by isopropyl alcohol, and quantified by spectrophotometer. The extracted total RNA was reversely transcribed into cDNA, and real-time QPCR amplification was performed by fluorescence QPCR instrument M_*X*_3005P using *β*-actin as an internal reference. All samples were converted to cDNA from RNA at the same concentration, and QPCR analysis was performed using cDNA at the same concentration. Two duplicate holes were made for each sample. The primers' sequences are as follows: PLUNC: the upstream primers are 5′-TTGAGTCCCACAGGTCTTGCAG-3′, the downstream primers are 5′-CTCCAGGCTTCAGGATGTCCA-3′. *β*-actin: the upstream primers are 5′-TGGCACCCAGCACAATGAA-3′, and the downstream primers are 5′-CTAAGTCATAGTCCGCCTAGAAGCA-3′. The reaction conditions of QPCR were 95°C. 30 s, 1 cycle; 95°C, 5 s; 60°C, 20 s, 40 cycle. The *C*_*t*_ value was calculated automatically after the reaction, and △*C*_*t*_ (△*C*_*t*_ = target gene and *C*_*t*_ = internal reference *C*_*t*_ was used to indicate the relative expression level of the PLUNC gene in different cell lines.

### 2.4. Bioinformatics Prediction and Probe Design

p-match (https://www.gene-regulation.com/pub/programs.html # pmatch) and Consite (https://mordor.cgb.ki.se/cgi-bin/CONSITE/consite/) were used for analysis and screened the transcription factors that are more likely to bind to C-1888T when the SNP is A or G, respectively. The binding core sequences of different transcription factors were obtained based on the binding site matrix information provided by TRANSFAC database. And the design method and principle of probe and mutant probe are referenced [13].

### 2.5. Electrophoretic Mobility Shift Assay (EMSA)

Nucleoproteins were extracted from CC and TT type cells at logarithmic growth stage referenced [14]. The protein concentration was measured by BCA method, and the oligonucleotide probes were labeled by biotin according to kit instructions. The nucleoproteins were bound to biotin-labeled probes (conventional response group), while a negative control group without nucleoprotein, a specific competition inhibition group with 200 times excessive cold probe, and a nonspecific competition inhibition group with 100 times excessive mutant probe were set at the same time. The DNA–nucleoprotein mixtures mentioned above were incubated at room temperature for 20 min and then added to 6% polyacrylamide gel pre-electrophoresis. After electrophoresis, the samples were transferred to a positively charged nylon film (380 mA, 40 min) and purple diplomatic strips. At last, the samples were exposed to x-ray film in a black box by detecting with a chemiluminescence nucleic acid detection kit.

### 2.6. Chromatin Immunoprecipitation (ChIP)

CC type NPC cells were cultured to 80% ∼ 90% fusion, and then, the culture medium was dumped. Cross-linking, lytic, and micrococcal nuclease digestion were performed according to the kit instructions, and the supernatant was collected by centrifugation, which contained the digested chromatin. 5 μL chromatin supernatant was taken as input and stored at −20°C for later use. The rest of the chromatin reacts with antibodies after 10-fold dilution. Different controls were set as follows: (1) the positive control: 500 μL diluted chromatin reacts with 10 μL anti-RNA polymerase II antibody; (2) the negative control: 500 μL diluted chromatin reacts with 1 μL normal rabbit IgG; (3) the sample reaction group: 500 μL diluted chromatin reacts with 1 μL rabbit antihuman EVI1 polyclonal antibody. The antibody–protein–DNA complex was precipitated with protein A/G, and DNA elution, decross-linking, and purification were performed after washing. Then, anti-RNA polymerase II ChIP DNA, IgG ChIP DNA, EVI1 antibody ChIP DNA, and input DNA were used as templates for PCR amplification, and the amplification system was 25 μL, the forward primer: 5′-AGGTGAGACAGTTAAGCTATTTGAT-3′, the reverse primer: 5′-AGGGGCCAAGAGATGAGACT-3′. The product was detected by 2% gel electrophoresis and then sequenced.

### 2.7. Statistical Analysis

SPSS 25.0 was used for analysis. The measurement data were expressed by means ± standard deviation, and the comparison of multiple means was performed by one-way ANOVA. *p* < 0.05 was considered significant.

## 3. Results

### 3.1. Screening for Three Genotypes of SNP Site

**Figure 1:**
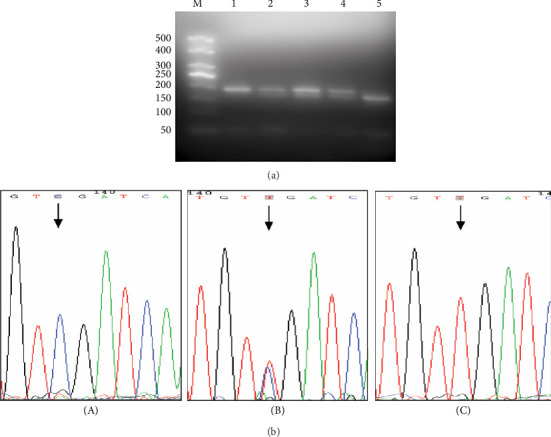
(a) PCR products were digested by FbaI enzyme. (b) Sequencing results. (A) represents 1 in (a) (homozygous CC type), (B) represents 2, 3, and 4 in (a) (heterozygous CT type), and (C) represents 5 in (a) (homozygous TT type).

NPC cell lines 5–8F, 6–10B, CNE1, and CNE2 were heterozygous CT type, SUNE1 was homozygous CC type, and C666-1 was homozygous TT type (the 22 bp band was gelated due to its small size). The direct sequencing results of PCR products were identical with those of PCR-RFLP interpretation analysis (Figure 1).

### 3.2. mRNA Expression of PLUNC Gene in Different Cell Lines

There were always sample wells failed to measure the value of *C*_*t*_ in the CNE1 cell line through repeated measurements, so the relevant statistical analysis was been abandoned. The relative expression amount △*C*_*t*_ value of PLUNC gene in different cell lines is shown in Table 1, and homogeneity of variance was tested by Levene (*F* = 2.395, *p*=0.120). The PLUNC gene expression of each cell line was significantly different. In addition, LSD multiple comparisons revealed significant differences in gene expression between CC and TT type cell lines (*p* < 0.001).

### 3.3. Bioinformatics Prediction and Probe Design and Synthesis

**Figure 2:**
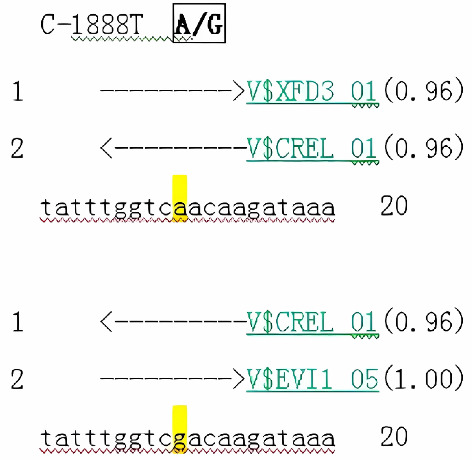
Prediction transcription factor binding site of C-1888T.

When the C-1888T site was G > A, different new transcription factor binding sites would be formed, and three candidate transcription factors --XFD3, EVI1, and CREL might bind to this site. Whether the site was A or G, CREL occurred in the same binding. However, XFD3 and EVI1 had a high probability of binding when the SNP locus was A and G, respectively (score was 0.96 and 1.00, respectively) (Figure 2).

**Figure 3:**
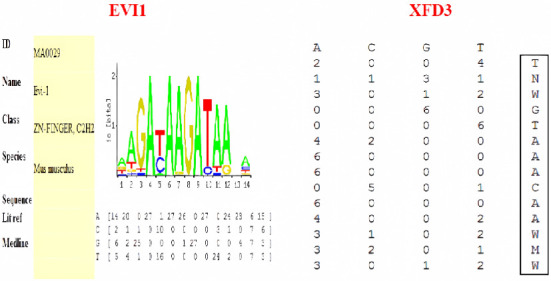
Matrix of transcription factor binding site.

According to the binding site matrix information provided by TRANSFAC database, the binding core sequences of XFD3 and EVI1 were TTGGTCAACAAGAT and CGACAAGATAA, respectively (Figure 3). The letter represented the binding site base sequence, and the numerical value after the letter represented the binding activity of each base. Based on the matrix information of each transcription factor, the mutant probe was designed using the substitution mutation method, which meant that the base with the highest binding activity was replaced with a base with low or no binding activity. The selection of each base was based on not producing new transcription factor binding sites. The detailed sequences are shown in Table 2.

### 3.4. EMSA

**Figure 4:**
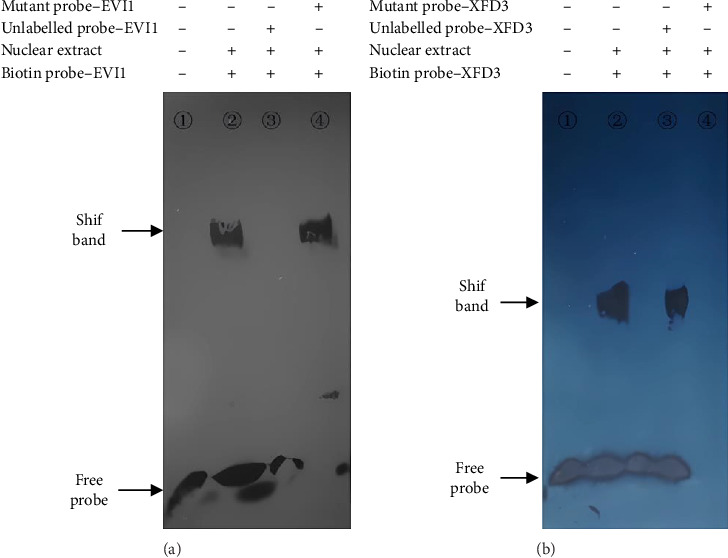
The specific binding of SNP site C-1888T in the promoter region of PLUNC gene to transcription.

When SNP C-1888T of PLUNC gene was A and G, respectively, the probe could bind to different transcription factors to form block bands and could be inhibited by specific competition. In contrast, the mutant probe could not inhibit the binding reaction (Figure 4).

### 3.5. ChIP Analysis

**Figure 5:**
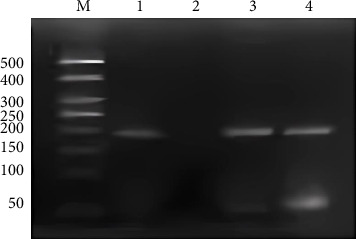
PCR analysis of EVI1 binding with PLUNC gene promoter in SUNE1 NPC cell line. M: marker, 1: input DNA, 2: IgG ChIP DNA, 3: EVI1 antibody ChIP DNA, 4: anti-RNA polymerase II ChIP DNA.

A 192 bp fragment of PLUNC gene promoter region containing the binding site of transcription factor EVI1 was amplified by PCR using DNA extracted from chromatin fragments immunoprecipitated by EVI1 antibody as the template. The results showed that a band appeared at 192 bp after anti-RNA polymerase II ChIP DNA, EVI1 antibody ChIP DNA, and input DNA amplification, while no bands appeared after the negative control IgG ChIP DNA amplification (Figure 5). That indicated the PLUNC gene promoter region has the binding site of EVI1.

## 4. Discussion and Conclusion

### 4.1. Discussion

The pathogenesis of NPC is a multistage, multipathway, and multimechanism process. No recognized specific oncogene of NPC was found except only some closely related genes. PLUNC gene, the candidate tumor suppressor gene, was first cloned in human nasopharyngeal tissue by Yao Kaitai's research group [6]. The expression of PLUNC gene showed relative tissue specificity. In situ hybridization results indicated that it was mainly expressed in the pseudolaminated ciliated columnar epithelium of the nasopharynx, trachea, and bronchus, and the gene expression gradually decreased along the respiratory tract from top to bottom [15]. And in the NPC biopsy tissues, the expression of PLUNC was decreased or not expressed [16]. However, the exact biological function of PLUNC gene is still unclear.

Our previous study on the SNP PLUNC gene confirmed an association between polymorphism in the promoter region of the gene and susceptibility to NPC. Three genotypes were identified by base mismatch, enzyme digestion, and sequencing. To understand whether this locus's polymorphism affected gene expression level, mRNA expression level of different cell lines was detected by fluorescence QPCR and the relationship between mRNA level of different genotypes and the polymorphism of this locus was analyzed. The Ct value has a logarithmic linear relationship with the initial copy number of the reaction template. The smaller Ct value indicated the more initial copy number of the gene and the higher expression of the gene; otherwise, the lower expression of the gene. The results of our study showed that the Ct values were highly measured by all cell lines, and the Ct values in CNE1 even failed to measure several times, which meant that the expression level of PLUNC gene in NPC cell lines was low. Previous literature reported that the expression of PLUNC gene in NPC tissues was extremely low by suppressive subtractive hybridization [17] or in situ hybridization. However, the PLUNC gene expression in cell mRNA level was verified this time, consistent with the previous results. The relative expression levels of PLUNC gene in different cell lines were significantly different (*p* ≤ 0.001). Further, multiple LSD comparisons showed that the expression levels of PLUNC gene in CC cell lines were significantly different from those in TT cell lines; the gene expression levels in CC cells were significantly lower than those in TT cell lines (*p* ≤ 0.001). Liu, He, and Wang [18] constructed two promoter-reporter gene (luciferase) expression vectors and detected luciferase activity. It was found that the activity of the 1888 C promoter was significantly lower than the basic promoter 1888 T of PLUNC, with a significant decrease of about 64.67%, and the difference between the two was statistically significant. Combined with these results, we hypothesized that C-C individuals were more susceptible to NPC because the polymorphic site down-regulates PLUNC gene expression by affecting its transcriptional regulation.

As a kind of protein molecule with a special structure and functions of regulating gene expression, transcription factor plays an important role in the occurrence, development, and metastasis of tumors [19]. Bioinformatics software predicted that transcription factor EVI1 had a high possibility of binding when the polymorphism site of C-1888T was G. To confirm whether the transcription factor can bind to the DNA fragment of the promoter region of PLUNC gene, the corresponding probes and a series of competitive reactions were designed using EMSA method. Using DNA extracted from chromatin fragment immunoprecipitated by EVI1 antibody as a template, the PLUNC gene promoter fragment containing the binding site of transcription factor EVI1 was successfully amplified by PCR. These results indicated that the binding of transcription factor EVI1 to DNA was specific and participated in regulating the PLUNC gene.

Transcription factor EVI1 is considered to be a proto-oncogene that plays a crucial role in tumorigenesis [20], and abnormal EVI1 expression is often observed in hematological malignancies [21] and some solid tumors [22–24]. Recent studies have shown that EVI1 may be associated with the occurrence of NPC [25], but there were few studies reported the biological role and potential mechanism of EVI1 in NPC. EVI1 contains two zinc finger protein domains, which can specifically recognize DNA [26]. Depending on its finger spatial structure, it extends into the groove of the DNA double helix and makes specific contact with DNA bases through the *α* helix [27]. It plays a vital role in gene expression regulation, cell differentiation, embryo development, and other aspects. The first zinc finger protein domain contained in EVI1 can inhibit TGF-*β* signaling [26, 28], and the latter is the most studied growth regulator, which can inhibit the proliferation of a variety of cell types and play a role in the occurrence and development of tumors. Lu et al. [29] reported that EVI1 might inhibit E-cadherin expression through the PTEN/PI3K/AKT pathway and ultimately affect epithelial–mesenchymal transition (EMT) and cancer stem cells (CSs) characteristics of NPC cells, which is closely related to the occurrence, development, invasion, and metastasis of NPC. Therefore, how to increase the susceptibility of the body after binding EVI1 and PLUNC gene promoter region, what are the specific regulatory mechanisms, and what signal transduction pathways lead to the proliferation of tumor cells need to be further clarified.

Transcription factor XFD3 has been identified in *Xenopus laevis*, now belonging to the Forkhead box (Fox) family [30]. Each member of the multigene family contains an evolutionarily conserved structure, the fork-head domain, which maintains the multifunction of embryonic stem cells, regulates apoptosis and cell cycle, regulates carbohydrate and lipid metabolism, and regulates immune regulation. The mutation and abnormal expression of the multigene family are related to developmental malformation, metabolic diseases, and neoplasms. According to the nomenclature, XFD3 is now called FoxA2a, and the coding sequence of the fork-head domain is highly homologous to Foxa2 in rodents and Foxa2 in humans [31, 32]. Hnf-3 *β* plays an important role in gastral differentiation, and ectopic HNF-3*β* can inhibit endoderm formation [33]. It is known that the epithelium of the respiratory tract, the epithelium of the digestive tract, the liver, and the pancreas are formed by endoderm differentiation. At present, studies on HNF-3*β*/Foxa2/Foxa2 were mainly focused on gastric cancer, liver cancer, pancreatic cancer, and other gastrointestinal tumors [34–36], lung cancer and other lung diseases [37], and the relationship with nasopharyngeal tumors had not been reported. Nasopharyngeal epithelium and lung epithelium both belong to the respiratory epithelium. Some researchers established mouse lung models exposed to cigarette smoke and found that Foxa2 and E-cadherin expression were down-regulated in airway epithelium. MAPK and Foxa2 mediated the development of squamous metaplasia in the lung of smoking rats, while MAPK inhibitors up-regulated Foxa2, leading to a decreased degree of lung squamous metaplasia [38]. In addition to the fork-head domain, the overall homology of proteins between rodents and *Xenopus* is about 70%. Therefore, further study needs to study whether there are differences in the final encoded proteins and functions and whether the signaling pathways involved are similar to those mentioned above. Although the EMSA results suggested that XFD3 could bind to the DNA fragment of the PLUNC gene promoter region, there was no suitable commercial XFD3 antibody at present, so the ChIP test was not performed to verify the EMSA results further. At the same time, human blood samples may be needed for future studies.

NPC demonstrates a familial aggregation phenomenon, with 10% of patients having a family history of tumors, suggesting that genetic susceptibility or predisposition may play a significant role in its pathogenesis. This study has shed light on some of the pathogenic factors of NPC through susceptibility research at polymorphic loci. However, it is crucial not to underestimate the impact of EB virus infection, environmental factors, chemical carcinogens, and carcinogens in the development of NPC. Moreover, the highest incidence of NPC was observed among the Chinese population, particularly the Cantonese, globally. With China being home to 56 ethnic groups, geographical, cultural, religious, social, political, or traditional factors may lead to distinct mutations due to isolation among different ethnicities, races, or groups. The study of gene transcription regulation is a complex network system, where in vitro and in vivo experiments may yield varying results, and differences in biological cell physiological states and environmental factors can influence experimental outcomes. Moving forward, our research will concentrate on refining and conducting in-depth analyses of various confounding factors. Increasing the sample size and encompassing multiple populations, ethnicities, and geographical regions will aid in providing a more comprehensive understanding of the relationship between the polymorphisms and susceptibility of PLUNC gene and NPC, as well as the mechanisms of transcriptional regulation.

### 4.2. Conclusion

In conclusion, the expression of the PLUNC gene, a tumor suppressor gene, was downregulated in NPC cell lines. This downregulation was more pronounced in CC type cell lines compared to TT type cell lines, indicating an elevated risk of NPC. We hypothesized that the C/T polymorphism at this locus led to differential binding of transcription factors, resulting in distinct transcriptional regulation processes that contributed to this risk. Through the utilization of bioinformatics tools for analysis, we validated the biological hypothesis using EMSA and ChIP assays to confirm the involvement of EVI1 in the transcriptional regulation of the PLUNC gene. This study sets the stage for further exploration of trans-acting factor regulatory mechanisms and biological functions of the PLUNC gene, with the ultimate aim of unraveling its functional mysteries and offering novel strategies for cancer prevention and treatment.

## Figures and Tables

**Table 1:** The △*C*_*t*_ value of PLUNC gene in different cell lines detected by quantitative real-time PCR.

Cell type	△*C*_*t*_ value ($\bar{x}\pm s$)	*F* value	*p* value
5–8F (CT type)	23.770 ± 1.308		
6–10B (CT type)	20.500 ± 0.209		
CNE2 (CT type)	23.903 ± 0.976		
SUNE1 (CC type)	25.163 ± 0.850⁣^∗^		
C666-1 (TT type)	18.100 ± 0.563⁣^∗^	33.844	< 0.001

LSD multiple comparison: ⁣^∗^*p* < 0.001.

**Table 2:** Transcription factor binding oligonucleotides and their mutated oligonucleotide sequences.

Probe	Sequence
EVI1-S	5′-GCTATTTGGTCGACAAGATAAACAGAGAAA-3′
EVI1-A	5′-CGATAAACCAGCTGTTCTATTTGTCTCTTT-3′
Mutant-EVI1-S	5′-GCTATTTGGTCCGGCCCGGGCACAGAGAAA-3′
Mutant-EVI1-A	5′-CGATAAACCAGGCCGGGCCCGTGTCTCTTT-3′
XFD3-S	5′-TAAGCTATTTGGTCAACAAGATAAACAGAG-3′
XFD3-A	5′-ATTCGATAAACCAGTTGTTCTATTTGTCTC-3′
Mutant-XFD3-S	5′-TAAGCTATCACAGTCGACCGGCAAACAGAG-3′
Mutant-XFD3-A	5′-ATTCGATAGTGTCAGCTGGCCGTTTGTCTC-3′

## Data Availability

The data supporting the findings of this study are included within the article.

## References

[1] Hashim N. A. N., Ramzi N. H., Velapasamy S. (2012). Identification of Genetic and Non-Genetic Risk Factors for Nasopharyngeal Carcinoma in a Southeast Asian Population. *Asian Pacific Journal of Cancer Prevention*.

[2] Tsao S. W., Tsang C. M., Lo K. W. (2017). Epstein-Barr Virus Infection and Nasopharyngeal Carcinoma. *Philosophical Transactions of the Royal Society B: Biological Sciences*.

[3] Liu Z., Chang E. T., Liu Q. (2017). Quantification of Familial Risk of Nasopharyngeal Carcinoma in a High-Incidence Area. *Cancer*.

[4] Paredes-Durán L. M., Del Barco-Morillo E., Baldeón-Conde M. J., Medina-Valdivieso S., Guillen-Sacoto M. C., Cruz-Hernández J. J. (2017). Familial Clustering of Nasopharyngeal Carcinoma in Non-Endemic Area. Report of Three Families. *Acta Otorrinolaringológica Española*.

[5] Shastry B. S. (2009). SNPs: Impact on Gene Function and Phenotype. *Methods in Molecular Biology*.

[6] He Z., Xie L., Xu L. (2000). Cloning of a Novel Gene Associated With Human Nasopharyngeal Carcinoma. *Chinese Science Bulletin*.

[7] Bingle C. D., Bingle L. (2000). Characterisation of the Human Plunc Gene, a Gene Product With an Upper Airways and Nasopharyngeal Restricted Expression Pattern. *Biochimica et Biophysica Acta*.

[8] Zhang B., Nie X., Xiao B. (2003). Identification of Tissue-Specific Genes in Nasopharyngeal Epithelial Tissue and Differentially Expressed Genes in Nasopharyngeal Carcinoma by Suppression Subtractive Hybridization and cDNA Microarray. *Genes, Chromosomes and Cancer*.

[9] He Y., Zhou G., Zhai Y. (2005). Association of PLUNC Gene Polymorphisms With Susceptibility to Nasopharyngeal Carcinoma in a Chinese Population. *Journal of Medical Genetics*.

[10] Wang S. (2007). *The Preliminary Study on Regulatory Mechanism of Human SPLUNC1 Expression*.

[11] Wang S., Yang K. T. (2009). Bioinformatics Analysis for Regulatory Elements of Tissue-Specific PLUNC Gene. *Journal of Tropical Medicine*.

[12] Liu B., Wang R., He Y. (2021). Functional Study of Polymorphism 1888 C > T in the Promoter Region of Human PLUNC Gene. *The American Journal of the Medical Sciences*.

[13] Green M. R., Sambrook J. (2013). *Molecular Cloning: A Laboratory Manual*.

[14] Verhoeven R. J., Tong S., Zhang G. (2016). NF-*κ*B Signaling Regulates Expression of Epstein-Barr Virus BART MicroRNAs and Long Noncoding RNAs in Nasopharyngeal Carcinoma. *Journal of Virology*.

[15] Wang S., Li W. L., Lü L. C., Yao K. T. (2016). Expressions of Short Palate, Lung and Nasal Epithelium Clone 1 in Different Human Tissues. *Nan Fang Yi Ke Da Xue Xue Bao*.

[16] Liu H., Zhang X., Wu J., French S. W., He Z. (2016). New Insights on the Palate, Lung, and Nasal Epithelium Clone (PLUNC) Proteins: Based on Molecular and Functional Analysis of its Homolog of YH1/SPLUNC1. *Experimental and Molecular Pathology*.

[17] Zhou Y., Zeng Z., Zhang W. (2008). Identification of Candidate Molecular Markers of Nasopharyngeal Carcinoma by Microarray Analysis of Subtracted cDNA Libraries Constructed by Suppression Subtractive Hybridization. *European Journal of Cancer Prevention*.

[18] Liu B., He Y., Wang S. (2009). The Construction of Luciferase Reporter Gene Vectors Containing Different Haplotypes DNA of Human PLUNC Gene Promoter Region. *Journal of Tropical Medicine*.

[19] Lambert M., Jambon S., Depauw S., David-Cordonnier M. H. (2018). Targeting Transcription Factors for Cancer Treatment. *Molecules*.

[20] Wieser R. (2007). The Oncogene and Developmental Regulator EVI1: Expression, Biochemical Properties, and Biological Functions. *Gene*.

[21] Hinai A. A., Valk P. J. (2016). Review: Aberrant *EVI* Expression in Acute Myeloid Leukaemia. *British Journal of Haematology*.

[22] Wu L., Wang T., He D., Li X., Jiang Y. (2019). EVI-1 Acts as an Oncogene and Positively Regulates Calreticulin in Breast Cancer. *Molecular Medicine Reports*.

[23] Queisser A., Hagedorn S., Wang H. (2017). Ecotropic Viral Integration Site 1, a Novel Oncogene in Prostate Cancer. *Oncogene*.

[24] Tanaka M., Ishikawa S., Ushiku T. (2017). EVI1 Modulates Oncogenic Role of GPC1 in Pancreatic Carcinogenesis. *Oncotarget*.

[25] Bei J. X., Li Y., Jia W. H. (2010). A Genome-Wide Association Study of Nasopharyngeal Carcinoma Identifies Three New Susceptibility Loci. *Nature Genetics*.

[26] Bard-Chapeau E. A., Gunaratne J., Kumar P. (2013). EVI1 Oncoprotein Interacts With a Large and Complex Network of Proteins and Integrates Signals Through Protein Phosphorylation. *Proceedings of the National Academy of Sciences of the United States of America*.

[27] Zhao N., Zhao F., Li Y. H. (2009). Advances in Research on Zinc Finger Protein. *Letters in biotechnology*.

[28] Deng X., Cao Y., Liu Y. (2013). Overexpression of Evi-1 Oncoprotein Represses TGF-*β* Signaling in Colorectal Cancer. *Molecular Carcinogenesis*.

[29] Lu Y., Liang Y., Zheng X., Deng X., Huang W., Zhang G. (2019). EVI1 Promotes Epithelial-to-Mesenchymal Transition, Cancer Stem Cell Features and Chemo-/Radioresistance in Nasopharyngeal Carcinoma. *Journal of Experimental & Clinical Cancer Research*.

[30] Kaestner K. H., Knochel W., Martinez D. E. (2000). Unified Nomenclature for the Winged Helix/Forkhead Transcription Factors. *Genes & Development*.

[31] Lef J., Dege P., Scheucher M., Forsbach-Birk V., Clement J. H., Knöchel W. (1996). A Fork Head Related Multigene Family Is Transcribed in *Xenopus laevis* Embryos. *International Journal of Developmental Biology*.

[32] Kaestner K. H. (2000). The Hepatocyte Nuclear Factor 3 (HNF3 or FOXA) Family in Metabolism. *Trends in Endocrinology and Metabolism*.

[33] Suri C., Haremaki T., Weinstein D. C. (2004). Inhibition of Mesodermal Fate by Xenopus HNF3*β*/FoxA2. *Developmental Biology*.

[34] Zhu C. P., Wang J., Shi B. (2015). The Transcription Factor FOXA2 Suppresses Gastric Tumorigenesis In Vitro and In Vivo. *Digestive Diseases and Sciences*.

[35] Chand V., Pandey A., Kopanja D., Guzman G., Raychaudhuri P. (2019). Opposing Roles of the Forkhead Box Factors FoxM1 and FoxA2 in Liver Cancer. *Molecular Cancer Research*.

[36] Wang B., Liu G., Ding L., Zhao J., Lu Y. (2018). FOXA2 Promotes the Proliferation, Migration and Invasion, and Epithelial Mesenchymal Transition in Colon Cancer. *Experimental and Therapeutic Medicine*.

[37] Jang S. M., An J. H., Kim C. H., Kim J. W., Choi K. H. (2015). Transcription Factor FOXA2-Centered Transcriptional Regulation Network in Non-Small Cell Lung Cancer. *Biochemical and Biophysical Research Communications*.

[38] Du C., Lu J., Zhou L. (2017). MAPK/FoxA2-Mediated Cigarette Smoke-Induced Squamous Metaplasia of Bronchial Epithelial Cells. *International Journal of Chronic Obstructive Pulmonary Disease*.

